# Emergent pancreatectomy for neoplastic disease: outcomes analysis of 534 ACS-NSQIP patients

**DOI:** 10.1186/s12893-020-00822-8

**Published:** 2020-07-27

**Authors:** Michael R. Driedger, Carlos A. Puig, Cornelius A. Thiels, John R. Bergquist, Daniel S. Ubl, Elizabeth B. Habermann, Travis E. Grotz, Rory L. Smoot, David M. Nagorney, Sean P. Cleary, Michael L. Kendrick, Mark J. Truty

**Affiliations:** 1 Division of Hepatobiliary and Pancreatic Surgery, Mayo Clinic, 200 First Street SW, Rochester, MN 55905 USA; 2 Robert D. and Patricia E. Kern Center for the Science of Health Care Delivery, Mayo Clinic, Rochester, MN USA; 3 Division of Health Care Research and Policy, Mayo Clinic, Rochester, MN USA

**Keywords:** Emergent, Pancreatectomy, Pancreas resection, Neoplasm, Cancer, Oncology

## Abstract

**Background:**

While emergent pancreatic resection for trauma has been previously described, no large contemporary investigations into the frequency, indications, and outcomes of emergent pancreatectomy (EP) secondary to complications of neoplastic disease exist. Modern perioperative outcomes data are currently unknown.

**Methods:**

ACS-NSQIP was reviewed for all non-traumatic pancreatic resections (DP – distal pancreatectomy, PD - pancreaticoduodenectomy, or TP- total pancreatectomy) in patients with pancreatico-biliary or duodenal-ampullary neoplasms from 2005 to 2013. Patients treated for complications of pancreatitis were specifically excluded. Emergent operation was defined as NSQIP criteria for emergent case and one of the following: ASA Class 5, preoperative ventilator dependency, preoperative SIRS, sepsis, or septic shock, or requirement of > 4 units RBCs in 72 h prior to resection. Chi-square tests, Fisher’s exact tests were performed to compare postoperative outcomes between emergent and elective cases as well as between pancreatectomy types.

**Results:**

Of 21,452 patients who underwent pancreatectomy for neoplastic indications, we identified 534 (2.5%) patients who underwent emergent resection. Preoperative systemic sepsis (66.3%) and bleeding (17.9%) were most common indications for emergent operation. PD was performed in 409 (77%) patients, DP in 115 (21%), and TP in 10 (2%) patients. Overall major morbidity was significantly higher (46.1% vs. 25.6%, *p* < 0.001) for emergent vs. elective operations. Emergent operations resulted in increased transfusion rates (47.6% vs. 23.4%, *p* < 0.001), return to OR (14.0% vs. 5.6%, *p* < 0.001), organ-space infection (14.6 vs. 10.5, *p* = 0.002), unplanned intubation (9.% vs. 4.1%, *p* < 0.001), pneumonia (9.6% vs. 4.2%, *p* < 0.001), length of stay (14 days vs. 8 days, *p* < 0.001), and discharge to skilled facility (31.1% vs. 13.9%). These differences persisted when stratified by pancreatic resection type. The 30-day operative mortality was higher in the emergent group (9.4%vs. 2.7%, *p* < 0.001) and highest for emergent TP (20%).

**Conclusion:**

Emergent pancreatic resection is markedly uncommon in the setting of neoplastic disease. Although these operations result in increased morbidity and mortality compared to elective resections, they can be life-saving in specific circumstances. The results of this large series of modern era national data may assist surgeons as well as patients and their families in making critical decisions in select cases of acutely complicated neoplastic disease.

## Background

Emergency pancreatic surgery is a rare event that is most commonly performed in the setting of blunt or penetrating abdominal trauma resulting in severe pancreatico-duodenal injury (perforation, bleeding, duct disruption) [1]. Emergent operative intervention is also occasionally warranted in the management of complications related to pancreatitis including ruptured pseudoaneurysms, bleeding pseudocysts, progressive multi-organ failure in the setting of severe necrotizing pancreatitis and walled-off pancreatic necrosis [2]. However outside of traumatic and pancreatitis complications, emergent pancreatectomy (EP) is rarely performed. Indications for EP in the setting of neoplastic disease is limited to life saving surgery for the control of hemorrhage, post-operative complications, malignant bowel/biliary perforations, or severe infections that have failed conservative management [2, 3].

In the elective setting, pancreas resections are being more commonly performed at high-volume centers with well-defined outcomes with anticipated perioperative morbidity and mortality, 32–38% and 0–5%, respectively [4, 5]. Utilizing the multi-center ACS-NSQIP dataset, we aimed to review the indications, patient demographics, and outcomes of patients requiring EP (DP – distal pancreatectomy, PD - pancreaticoduodenectomy, or TP- total pancreatectomy) in the setting of non-traumatic, non-pancreatitis, neoplastic disease where the modern perioperative outcomes of these extremely uncommon procedures are currently unknown [6]. Given the rarity of these procedures, outcomes of emergent pancreatic resection in this specific setting of non-traumatic, neoplastic disease is limited to small institutional case series. Previous reports suggest that the mortality rate for EP is between 40 and 53% and thus prohibitive [2, 7]. Furthermore, there are no reports comparing emergent outcomes between specific pancreatectomy types (PD, DP and TP) outside of trauma patients.

## Methods

Data was queried from the American College of Surgeons National Surgical Quality Improvement Program (ACS-NSQIP) database from 2005 to 2013. ACS-NSQIP collects data on 305 variables including 30-day morbidity and mortality for patients undergoing surgical procedures. All patients with a Current Procedural Terminology (CPT) code for Pancreaticoduodenectomy (PD, 48150, 48,153, 48,152, and 48,154), Distal Pancreatectomy (DP, 48140, 48,145, 48,146, and 48,999), or Total Pancreatectomy (TP, 48155 and 48,160) were queried. Only patients with an International Classification of Diseases 9th Revision (ICD-9) code for pancreas cancer (157.x, excluding 157.4), duodenal neoplasm (152, 152.0, 211.2, 230.7, 235.2), neuroendocrine tumor (157.4; 209.29; 209.3; 209.30; 209.69; 211.7), and bile duct and ampullary neoplasm (156.1; 156.2; 156.8; 156.9; 211.5; 211.6; 230.8; 235.3) were included in the analysis. Patients with an ICD-9 code for pancreatitis (577.0–577.9) or a procedure code for resection or debridement of the pancreas for acute necrotizing pancreatitis (48105) were excluded to minimize confounding ambiguity. We specifically excluded patients that underwent emergent re-operation after recent previous pancreatectomy. Three patients had both a DP and PD and were excluded from the analysis. Operations were considered to be emergent if the case was coded as emergent per ACS-NSQIP or any of the following: American Society of Anesthesiologists (ASA) Class 5, preoperative ventilator dependency, preoperative Systemic Inflammatory Response Syndrome (SIRS), sepsis, or septic shock, or requirement of > 4 units RBCs in 72 h prior to resection. Pre-operative mechanical ventilation was utilized as a surrogate indicator for EP given the ICU status of patient and obvious contraindication to elective oncologic resection. SIRS, sepsis and septic shock were defined according to the 2001 international sepsis definitions conference [8]. All other concurrent procedures were included. It is important to note that ACS-NSQIP does not include trauma patients and therefore these patients are not included in the analysis.

The primary end points of this study were 30-day morbidity and mortality. Major postoperative morbidity was defined as: cardiac arrest, myocardial infarction, stroke/CVA with deficits, wound disruption, deep incisional surgical site infection (SSI), organ space SSI, sepsis or septic shock, unplanned intubation, postoperative ventilator requirement exceeding 48 h, pneumonia, acute renal failure, progressive renal insufficiency, DVT/thrombophlebitis, pulmonary embolism, and unplanned return to OR. While there exists no ACS-NSQIP code for postoperative pancreatic fistula (POPF), organ-space surgical infection is often utilized as a proxy for this development and was thus included [9]. Of note, organ space infections are not limited to pancreatic fistula and may include intra-abdominal abscesses or other infected abdominal fluid collections not associated with POPF. Minor morbidity included superficial SSI and UTI. SSIs are classified as superficial, organ space, or deep/incisional. Postoperative transfusion was defined differently during the years the database was queried. From 2005 to 2009 postoperative transfusion was defined as units of blood given following the index operation. From 2010 to 2013 the definition was expanded to include both intraoperative and postoperative transfusion requirements. Patient discharge disposition was collected by NSQIP beginning in 2011 and therefore data is only available from this time point onward. Only live discharges were included in reporting discharge disposition. Chi-square tests, Fisher’s exact tests, student’s t-tests, Kruskal-Wallis tests, and rank sum tests were performed to compare demographic characteristics and postoperative outcomes between groups with a *p* < 0.05 being considered significant. The Cochran-Armitage test for trend *p*-values (two-sided) was used to compare annual outcomes over the course of the study period. Only 5 covariates could be utilized in the multivariable modeling as there were only 50 patients with the outcome of interest (30-day mortality). Given the changes in transfusion data and limited availability of preoperative chemotherapy and radiation therapy at all participating NSQIP centers in the later years of our study, these were excluded from multivariate modeling. All statistical analysis was performed using SAS Version 9.3 (SAS Institute Inc., Cary NC).

## Results

### Demographics

During the study period 21,452 patients underwent pancreatectomy for non-traumatic, non-pancreatitis, neoplastic disease indications. Of these, 534 patients (2.5%) underwent an EP based upon our defined parameters. Preoperative systemic sepsis (66.3%) and transfusion-dependent hemorrhage (17.9%) were the most common indications for emergent pancreatectomy (Table 1). Forty-seven (9%) patients had greater than one indication for an emergent operation. The most common neoplastic pathologies were pancreatic cancer, duodenal neoplasms and neuroendocrine tumors. The distribution of pancreatectomy types performed were as follows; PD (*n* = 409, 76.6%), DP (*n* = 115, 21.5%), and TP (*n* = 10, 1.9%). Mean age of all patients undergoing an EP was 64.7 ± 12.7 years. The median age in the PD and TP groups (68 and 67 years, respectively) was greater than those who underwent a DP (60 years) (*p* < 0.001). Overall, 48.7% of the EP cases were male gender with similar distributions between the different types of pancreatectomy (PD 47.9% male, DP 50.4% male, and TP 60.0% male, *p* = 0.69). Median BMI was similar across all groups, 26.4 in PD, 26.8 in DP, and 22.9 in TP (*p* = 0.22). Patients undergoing emergent TP or PD had a higher preoperative bilirubin levels (median 1.8 mg/dl and 1.5 mg/dl) compared to those undergoing DP (0.5 mg/dl), (*p* < 0.001). There were no differences in preoperative functional status, transfusion requirements, delivery of chemotherapy and/or radiotherapy in the preceding 30 days between pancreatectomy types.

**Table 1:** Demographics of all Emergent Pancreatectomy Patients

Demographics	All (*n* = 534)	PD (*n* = 409)	DP (*n* = 115)	TP (*n* = 10)	*p*-value (across groups)	*p*-value (DP vs. PD)
Male, no. (%)	260 (48.7)	196 (47.9)	58 (50.4)	6 (60.0)	0.69	0.63
Age, yrs., median	66	68	60	67	< 0.001	< 0.001
BMI, median	26.5	26.4	26.8	22.9	0.22	0.79
Preoperative bili, median mg/dL	1	1.5	0.5	1.8	< 0.001	< 0.001
Preoperative sepsis, no. (%)	353 (66.4)	288 (70.8)	57 (49.6)	8 (80.0%)	< 0.001	< 0.001
> 4 units PRBC in 72 h before surgery (2005–2012), no. (%)	72 (17.9)	53 (17.2)	18 (20.7)	1 (12.5)	0.7	0.43
> 10% loss body weight in last 6 months, no. (%)	118 (22.1)	98 (24.0)	16 (13.9)	4 (40.0)	0.028	0.021
Functional status, no. (%)					0.095	0.58
Independent	483 (91.0)	369 (90.7)	107 (93.9)	7 (70.0)		
Partially dependent	37 (7.0)	28 (6.9)	6 (5.3)	3 (30.0)		
Totally dependent	11 (2.1)	10 (2.5)	1 (0.9)	0 (0.0)		
Chemo/Radiotherapy in ≤30 days pre-op, no. (%)	17 (4.7)	12 (4.2)	3 (4.2)	2 (25.0)	1	1

*Abbreviations*: *PD* pancreaticoduodenectomy, *DP* distal pancreatectomy, *TP* total pancreatectomy, *Bili* bilirubin

### Emergent vs elective overall outcomes

Table 2 contrasts outcomes between emergent and elective cases involving pancreatic resection. Unsurprisingly, overall morbidity was greater in the EP group (53.6% vs 32.7%, *p* < 0.001). Furthermore, major complications were experienced in 46.1% of emergent cases compared to 25.6% elective patients (*p* < 0.001). No difference was demonstrated in the SSI rate between the emergent and elective cohorts (25.1% vs 19.6%, *p* = 0.7). However, when stratified by SSI subtype, organ-space infection rates were greater in those undergoing an EP versus elective pancreatectomy (14.6% vs 10.5%, *p* < 0.001). Unplanned intubation and return to the OR rates were also greater (*p* < 0.001). With the increased morbidity associated with EP, median LOS was prolonged by 6 days (14 vs 8 days, *p* < 0.001) and more patients required discharge to a skilled nursing facility for rehabilitation (31.1% vs 13.9%, *p* < 0.001). Lastly, 30-day mortality for the EP cohort was significantly increased at 9.4% compared to 2.7% in the elective cohort (*p* < 0.001).

**Table 2:** Perioperative Outcomes: Emergent vs. Elective Pancreatectomy

Perioperative Outcome	All Emergent Pancreatectomies (*n* = 534)	All Elective Pancreatectomies (*n* = 20,918)	*p*
OR Time min, median	334	322	0.43
Blood Transfusion, no. (%)	149 (47.6)	3189 (23.4)	< 0.001
Any Complication, no. (%)	286 (53.6)	6843 (32.7)	< 0.001
Major Complication, no. (%)	246 (46.1)	5349 (25.6)	< 0.001
Unplanned intubation, no. (%)	48 (9.0)	864 (4.1)	< 0.001
Pulmonary embolism, no. (%)	5 (0.9)	260 (1.2)	0.53
Pneumonia, no. (%)	51 (9.6)	870 (4.2)	< 0.001
SSI, no. (%)	41 (7.7)	1702 (8.1)	0.70
Organ-space infection, no. (%)	78 (14.6)	2187 (10.5)	0.002
Urinary tract infection, no. (%)	47 (8.8)	970 (4.6)	< 0.001
Return to OR, no. (%)	75 (14.0)	1161 (5.6)	< 0.001
LOS days, (median)	14	8	< 0.001
Discharge to home, no. (%)^a^	162 (68.9)	9686 (86.1)	< 0.001
30-day Mortality, no. (%)	50 (9.4)	557 (2.7)	< 0.001

^a^variable added in 2011, no. (%) of available patient data

*Abbreviations*: *SSI* surgical site infection, *LOS* length of stay

### Comparative outcomes by Pancreatectomy type (emergent vs. elective)

The comparative outcomes between emergent and elective cases stratified by pancreatectomy type are demonstrated in Table 3. Median operative time was similar between emergent and elective cases for PD, DP and TP. The requirement for perioperative blood transfusion was increased in the emergent PD (EPD) and DP (EDP) groups compared to the elective cohorts (PD 49.8% vs 26.4%, DP 38.8% vs 14.9%, *p* < 0.001). No difference for perioperative transfusion in the emergency TP (ETP) group was seen (66.7% vs 40.2%, *p* = 0.57). The major complication rate was greater in emergent vs elective PD (48.7% vs. 27.7%, *p* < 0.001). The same held true for emergent vs elective DP (38.3% vs. 20.3%, *p* < 0.001), while no difference existed between patients undergoing emergent vs elective TP (30.0% vs. 29.2%, *p* = 1). For the EPD group, organ-space infection rates were higher than those in the elective cohort (15.6% vs. 11.2%, *p* < 0.005). For both DP and TP, no difference was demonstrated for organ space infection rate (DP 11.3% vs. 8.9%, *p* = 0.37 and TP 10% vs. 9.3%, *p* = 1). Mean LOS was prolonged for all pancreatectomy subgroups, but this only reached statistical significance for PD (15 vs 9 days, *p* < 0.001) and DP (9 vs 6 days, *p* < 0.001). Furthermore, EPD and EDP patients required transfer to a skilled nursing facility at rates greater than those in the elective groups (PD 35.6% vs 16.7%, *p* < 0.001, DP 17.0% vs 7.3%, *p* = 0.015). Lastly, 30-day mortality was 10.3% in the EPD group vs. 3.2% in the elective cohort (*p* < 0.001). For the DP group, mortality was also higher in the emergent vs elective group (5.2% vs. 1.2%, *p* < 0.001). While there was no statistical difference in the 30-day mortality rate between the TP cohorts, a trend towards increased mortality was seen (20% vs. 5.6% *p* = 0.11).

**Table 3:** Perioperative Outcomes: Emergent vs. Elective Pancreatectomy

Perioperative Outcome	Emergent PD (*n* = 409)	Elective PD (*n* = 14,409)	*p*	Emergent DP (*n* = 115)	Elective DP (*n* = 6077)	*p*	Emergent TP (*n* = 10)	Elective TP (*n* = 432)	*p*
OR Time min, median	361	358	0.46	208	216	0.49	378	375	0.92
Blood Transfusion, no. (%)	121 (49.8)	2486 (26.4)	< 0.001	26 (38.8)	588 (14.9)	< 0.001	2 (66.7)	115 (40.2)	0.57
Any Complication, no. (%)	230 (56.2)	5187 (36.0)	< 0.001	53 (46.1)	1509 (24.8)	< 0.001	3 (30.0)	147 (34.0)	1.0
Major Complication, no. (%)	199 (48.7)	3989 (27.7)	< 0.001	44 (38.3)	1234 (20.3)	< 0.001	3 (30.0)	126 (29.2)	1.0
Unplanned intubation, no. (%)	4 (11.0)	718 (5.0)	< 0.001	2 (1.7)	125 (2.1)	1.0	1 (10)	21 (4.9)	0.4
PE, no. (%)	2 (0.5)	148 (1.0)	0.45	3 (2.6)	111 (1.8)	0.47	0 (0.0)	1 (0.2)	1.0
Pneumonia, no. (%)	40 (9.8)	651 (4.5)	< 0.001	10 (8.7)	200 (3.3)	0.002	1 (10.0)	19 (4.4)	0.37
SSI, no. (%)	35 (8.6)	1440 (10.0)	0.34	6 (5.2)	237 (3.9)	0.47	0 (0.0)	25 (5.8)	1.0
Organ-space infection, no. (%)	64 (15.6)	1607 (11.2)	0.005	13 (11.3)	540 (8.9)	0.37	1 (10.0)	40 (9.3)	1
UTI, no. (%)	36 (8.8)	711 (4.9)	< 0.001	11 (9.6)	237 (3.9)	0.002	0 (0.0)	22 (5.1)	1.0
Return to OR, no. (%)	64 (15.6)	910 (6.3)	< 0.001	10 (8.7)	215 (3.5)	0.003	1 (10.0)	36 (8.3)	0.59
LOS days, (median)	15	9	< 0.001	9	6	< 0.001	15	9	0.59
Discharge to home, no. (%)^a^	116 (64.4)	6448 (83.3)	< 0.001	44 (83.0)	3031 (92.7)	0.015	2 (100.0)	207 (86.3)	0.57
30-day Mortality, no. (%)	42 (10.3)	458 (3.2)	< 0.001	6 (5.2)	75 (1.2)	< 0.001	2 (20.0)	24 (5.6)	0.11

^a^variable added in 2011, no. (%) of available patient data

*Abbreviations*: *PE* pulmonary embolism, *UTI* urinary tract infection

### Comparative outcomes by Pancreatectomy type in the emergent cohort

Differences in perioperative outcomes within the EP cohort by pancreatectomy type are shown in Table 4. There was a trend for major complications to be greater in the PD group (48.7%), compared to DP (38.3%) and TP (30%) (*p* = 0.08 across all groups, DP vs. PD *p* = 0.12). SSIs of any type were the highest in the PD group (27.1%) compared to DP (19.1%) and TP (10.0%) (*p* = 0.04 across all groups, DP vs. PD *p* = 0.08). No difference in organ-space infection rates in the PD (15.6%) and DP groups (11.3%) groups compared that experienced in the TP group (10%) (*p* = 0.47 across all groups, DP vs. PD *p* = 0.25). Perioperative blood transfusion requirements were the highest in the TP group (66.7%) compared to 49.8% in PD and 38.8% in DP (*p* < 0.001). Median LOS was similar for the TP group (15 days) and PD group (15 days) compared to the DP group (9 days) (*p* < 0.001 across all groups, DP vs. PD *p* < 0.001). Thirty-day mortality was greatest in the TP group (20%) compared to 10.3% in PD and 5.2% in DP (*p* = 0.13 across all groups, DP vs. PD *p* = 0.097).

**Table 4:** Perioperative Outcomes: Emergent Pancreatectomy by Type

Perioperative Outcome	PD (*n* = 409)	DP (*n* = 115)	TP (*n* = 10)	*p*-value (across 3 groups)	*p*-value (DP vs. PD)
OR Time min, median	361	208	378	< 0.001	< 0.001
Blood Transfusion, no. (%)	121 (49.8)	26 (38.8)	378	0.47	0.25
Any Complication, no. (%)	230 (56.2)	53 (46.1)	2 (66.7)	0.59	0.8
Major Complication, no. (%)	199 (48.7)	44 (38.3)	3 (30.0)	0.24	0.11
Unplanned intubation, no. (%)	4 (11.0)	2 (1.7)	3 (30.0)	0.003	0.001
Pulmonary embolism, no. (%)	2 (0.5)	3 (2.6)	1 (10)	0.16	0.073
Pneumonia, no. (%)	40 (9.8)	10 (8.7)	0 (0.0)	0.94	0.73
SSI, no. (%)	35 (8.6)	6 (5.2)	1 (10.0)	0.12	0.081
Organ-space infection, no. (%)	64 (15.6)	13 (11.3)	0 (0.0)	0.32	0.24
Urinary tract infection, no. (%)	36 (8.8)	11 (9.6)	1 (10.0)	0.87	0.8
Return to OR, no. (%)	64 (15.6)	10 (8.7)	0 (0.0)	0.15	0.059
LOS days, (median)	15	9	15	< 0.001	< 0.001
Discharge to home, no. (%)^a^	116 (64.4)	44 (83)	2 (100.0)	0.022	0.011
30-day Mortality, no. (%)	42 (10.3)	6 (5.2)	2 (20.0)	0.13	0.084

^a^variable added in 2011, no. (%) of available patient data

### Additional outcomes

On univariate analysis, predictors of 30-day mortality included; age, BMI, jaundice, sepsis, preoperative transfusion > 4 units in the 72 h prior to surgery, impaired functional status and delivery of systemic chemotherapy or radiation therapy within preceding 30 days. Gender as well as weight loss exceeding 10% in the 6 months prior to EP did not influence mortality (Table 5). Multivariate modeling was employed to further assess predictors of 30-day mortality (Table 6). Variables included; age, BMI, preoperative sepsis and functional status. Interestingly, the only pre-operative factor that statistically influenced 30-day mortality was functional status (*p* = 0.02, OR 2.60, 95% CI 1.16–5.84).

**Table 5:** Univariate Analysis for 30-Day Mortality

Variable	p*
Gender	0.28
Age (18–69 vs > 70)	**< 0.001**
BMI (< 30 vs > 30)	**0.015**
Elevated Preoperative bilirubin	**0.012**
Preoperative sepsis	**0.007**
> 4 units packed red blood cells in 72 h before surgery	**0.003**
> 10% loss body weight in last 6 months	0.29
Functional status	**< 0.001**
Chemo/Radiotherapy in ≤30 days pre-op	**0.003**

*Abbreviation*: *BMI* Body mass index

*Chi-square significance, 2-sided

**Table 6:** Multivariate Analysis for 30-Day Mortality

Variable	OR for 30-Day Mortality	95% CI	*p*
Age (18–69 vs > 70)	1.73	0.93–3.22	0.086
BMI (< 30 vs > 30)	0.56	0.24–1.31	0.18
Preoperative sepsis			
No vs SIRS	0.93	0.46–1.89	0.84
No vs Sepsis/Septic Shock	1.10	0.44–2.75	0.84
Functional status (Partial/Total Dependence vs Independent)	**2.60**	**1.16–5.84**	**0.020**

*Abbreviations*: *OR* odds ratio, *95% CI* 95% confidence interval, *SIRS* systemic inflammatory response syndrome

The rates of morbidity (minor, major and total), return to OR during index hospital stay and 30-day mortality were categorized by year during the period of data analysis (2005–2013). Specifically, no difference was appreciated for total morbidity (*p* = 0.47) and 30-day mortality (*p* = 0.93) over the study period (Table 7). There was however a statistical difference appreciated for return to OR (*p* < 0.001), in that the rate of unplanned return to the OR decreased from 2005 to 2013.

**Table 7:** Operative Outcomes by Year

Perioperative Outcome	All (*n* = 534)	2005 (*n* = 8)	2006 (*n* = 52)	2007 (*n* = 48)	2008 (*n* = 49)	2009 (*n* = 64)	2010 (*n* = 77)	2011 (*n* = 105)	2012 (*n* = 64)	2013 (*n* = 67)
Morbidity	286 (53.6)	6 (75.0)	36 (69.2)	27 (56.3)	19 (38.8)	31 (48.4)	43 (55.8)	50 (47.6)	31 (48.4)	43 (64.2)
Return to OR, no. (%)	75 (14.0)	2 (25.0)	15 (28.8)	8 (16.7)	6 (12.2)	9 (14.1)	12 (15.6)	12 (11.4)	6 (9.4)	5 (7.5)
30-day Mortality, no. (%)	50 (9.4)	0 (0.0)	7 (13.5)	4 (8.3)	2 (4.1)	7 (10.9)	6 (7.8)	14 (13.3)	3 (4.7)	7 (10.4)

Forty-four patients underwent emergent pancreatic resection in the setting of benign disease (15 PD, 29 DP, 0 TP). While total morbidity and 30-day mortality rates were similar when compared to those with underlying invasive disease, two differences emerged. Patients with malignant disease experienced a higher rate of postoperative transfusion (49.5% vs. 20% *p* = 0.011) and a greater duration of median length of stay (14 days vs. 9 days, *p* = 0.04).

## Discussion

To our knowledge this is the most contemporary analysis and first nationwide evaluation of perioperative outcomes for patients undergoing EP for complications resulting from a neoplastic process. Our dataset excluded those whose operative indication arose from trauma or pancreatitis. Although emergent completion pancreatectomy has been reported for patients requiring control of sepsis or bleeding after initial elective partial pancreatectomy, these patients were also specifically excluded from analysis. The principal focus of the investigation was to analyze outcomes in patients undergoing EP in which a neoplastic process was the primary pathology. Furthermore, all previous small case series evaluating outcomes for EP included only PD, while comparative outcomes for various pancreatectomy types such as DP or TP have not been reported [2, 7].

Only 2.5% of all pancreatic resections performed for neoplastic indications during the study period occurred in an emergent setting, demonstrating the rarity of this occurrence. Indications included control of sepsis and hemorrhage. As expected, 30-day morbidity and mortality in patients undergoing emergent pancreatic resections (PD, DP or TP) were significantly higher than the non-emergent/elective cases. This correlates with previously published perioperative elective outcomes [9–11]. When stratifying postoperative outcomes by specific procedure, TP in the elective and emergent setting had similar findings with respect to major morbidity. This is best explained by the influence of limited patient numbers on statistical analysis.

Regarding complications specific to pancreatectomy, organ-space surgical infection was queried as this served as a surrogate for the rate of POPF. In an ACS-NSQIP study by Tseng et al. [9], the rate of organ space surgical infection after elective DP was 8.1%, which was comparable to the summation of incidence of pancreatic fistula (5%) and intra-abdominal abscesses (4%) in a review by Lillemoe et al. [12]. The modern incidence of clinically significant pancreatic fistulae as defined by the International Study Group of Pancreatic Surgery is variable and dependent on numerous factors and patient characteristics. Rates in the literature range from 5% to greater than 30% [13–17]. Utilizing organ-space surgical infection as a surrogate may underestimate the true incidence of pancreatic fistula but is nonetheless a useful metric to consider. The organ-space surgical infection rate following emergent PD and DP was elevated and is therefore suggestive of increased POPF.

The data presented here are unique as very few previous publications on emergent pancreatic resection exist and are solely single institution reviews. In an earlier series of over 400 pancreatic head resections, the prevalence of emergency non-traumatic pancreatoduodenectomy was 1% [7]. This unique indication resulted in substantially increased morbidity and mortality. A more recent review combined data from a single institution with previously published outcomes in the literature. The overall rate for non-traumatic pancreaticoduodenectomy was 0.3–3% which aligns with our current national series. In that analysis, surgical morbidity (84%) and mortality (20%) was significantly higher when compared to elective cases. Interestingly, perforation as the operative indication carried the highest risk of morbidity and mortality [18]. In contrast, a smaller series of 300 patients undergoing pancreaticoduodenectomy revealed only 6 (2%) emergent cases. The indications for surgical intervention were perforation, bleeding and postoperative complications. No significant difference in surgical complication rates or mortality due to the emergent nature of the resection was idenitified [2]. Our data aligns with previously published results, confirming both the rarity of the event and associated elevation in perioperative morbidity. Given the increased power of our dataset and reasonable national sampling, the specific risks are likely well represented. Despite the increased morbidity and mortality associated with an EP in the setting of an underlying neoplastic pathology, the heightened risk is not necessarily prohibitive. Careful and appropriate patient selection must guide surgical decision making and take into account a multitude of factors. This dataset will significantly inform patients, families and clinicians when these unique clinical situations arise.

Of note, patients undergoing EP experienced an increased length of hospital stay of 6 days and nearly one-in-four were transferred to an alternate care facility to facilitate rehabilitation. This has important implications on health care expenditure and resource allocation. Additionally, if a decision is made to undergo emergent pancreatectomy in the setting of cancer, the patient must fully understand the long-term effects on quality of life and the ability to return to preoperative or acceptable levels of functioning. In the elective setting, postoperative PD patients have a quality of life and independent functioning that is equivalent to, or approaches, that in the general population [19, 20]. It is important to note that NSQIP database is limited to 30-day outcomes. Therefore, only short-term outcome metrics were available for the present study. While long term outcomes, cancer-specific outcomes, and quality of life data were not available, this would be an interesting area of further inquiry and certainly warrants patient discussion.

Functional status emerged as the sole predictor of 30-day mortality on multivariate modeling in the setting of emergent pancreatectomy. This finding is congruent with growing surgical literature highlighting the clinical significance and prognostic performance of this specific patient metric [21–24]. However, physician assessment of functional status is of uncertain accuracy. An international, prospective cohort study published in 2018 demonstrated that subjective assessment of functional status was 19.2% sensitive and 94.7% specific for identifying the inability to attain four metabolic equivalents during cardiopulmonary exercise testing [25]. Importantly, in the setting of emergent surgery, an accurate and detailed assessment of the patient’s cardiovascular and global risk profile is not possible. Therefore, clinicians must utilize their clinical acumen and experience to create an individualized assessment of risk and benefit. This will certainly incorporate the patient’s functional status, comorbidity profile, oncologic history and predicted outcomes, coupled with the specifics of the clinical situation mandating emergent operative intervention.

The significant influence of both surgeon and hospital volume on patient outcome is unaccounted for in this analysis. The true indications for EP in the setting of neoplastic disease are rare and should ideally be only considered and performed in high volume referral centers. It is also unclear if any patients could have been temporized utilizing interventional endoscopic or radiologic procedures. Notably, the rate of unplanned return to the OR decreased from 2005 to 2013. Unfortunately, the factors contributing to this finding cannot be accurately determined with the available dataset, but may reflect continued improvements in critical care and advancements in interventional and endoscopic procedures.

The present study has several limitations. First and foremost is the lack of detailed case data available from the NSQIP database. This included the absence of pancreas-specific outcomes in the database, namely POPF. In addition, the rarity of TP in the emergent setting also leaves the study underpowered to better compare outcomes of this cohort to those undergoing emergent DP or TP. The emergent nature of the pancreatectomy was inferred if coded as such within the database or if the patient was labelled an ASA class 5, mechanically ventilated, experienced preoperative systemic sepsis (SIRS, sepsis or septic shock), or was transfused greater than 4 units in the preceding 72 h. Data was unavailable for the specific indications for emergent pancreatic resection such as bleeding tumor, duodenal perforation, etc. Although preoperative systemic sepsis was the primary indication for emergent pancreatectomy, the specific cause of this sepsis was uncertain. NSQIP does not code for type of hospital setting (Academic, Private, Rural), or type of surgeon (HPB, general or trauma surgeon), so it is impossible to tell by who and in which setting these operations were performed. The strengths of this study include a large national dataset with straightforward and sound statistical comparisons.

## Conclusion

Emergent, non-traumatic, non-pancreatitis, pancreatic resection in the setting of neoplastic disease results in significantly higher mortality and morbidity compared to elective pancreatic resections. Although emergent surgery carries substantial risk, in selected cases operative intervention should be strongly considered. The results of this large series of modern national data will inform the challenging clinical decision making that surrounds these rare and unanticipated patient presentations.

## Data Availability

The datasets used and/or analysed during the current study are available from the corresponding author on reasonable request. The Participant Use Data File (PUF) is a Health Insurance Portability and Accountability Act (HIPAA)-compliant data file containing cases submitted to ACS NSQIP. The PUF is provided to employees of ACS NSQIP-participating hospitals. To request access to the PUF, contact ACS NSQIP staff. https://www.facs.org/quality-programs/acs-nsqip/participant-use/puf-form

## Abbreviations

EP: Emergent Pancreatectomy
(E) PD: (Emergent) Pancreaticoduodenectomy
(E) DP: (Emergent) Distal Pancreatectomy
(E) TP: (Emergent) Total Pancreatectomy
ACS-NSQIP: American College of Surgeons National Surgical Quality Improvement Program
SIRS: Systemic Inflammatory Response Syndrome
ASA: American Society of Anesthesiologists
